# A novel and highly efficient purification procedure for native human dipeptidyl peptidase 3 from human blood cell lysate

**DOI:** 10.1371/journal.pone.0220866

**Published:** 2019-08-07

**Authors:** Paul Kaufmann, Matthias Muenzner, Mandy Kästorf, Karine Santos, Tobias Hartmann, Anke Dienelt, Linda Rehfeld, Andreas Bergmann

**Affiliations:** 1 Sphingotec GmbH, Hennigsdorf, Germany; 2 Sphingotec Therapeutics GmbH, Hennigsdorf, Germany; Instituto Butantan, BRAZIL

## Abstract

Dipeptidyl amino-peptidase 3 (DPP3) is an aminopeptidase involved in peptide degradation, including hormone peptides as angiotensin II and enkephalins. DPP3 plasma activity increases in septic patients and correlates with mortality risk. However, the exact physiological role of DPP3 remains unclear and animal studies are necessary to reveal the function of DPP3 *in vivo*. To this demand, we developed a two-step purification procedure for isolation of native human DPP3 from blood cell lysate (BCL) that is suitable for *in vivo* applications. With the use of monoclonal antibodies coupled to beads in combination with an ion-exchange chromatography, we recovered 68% of human DPP3 activity from BCL with a purity of ≥ 95%. Purified human DPP3 was assayed for activity and protein concentration using recently published DPP3-activity- and immunoassays. Additionally, protein stability and storage in relevant buffers were tested. Our results provide a promising strategy for fast and efficient isolation of human DPP3. The purified human DPP3 represents the native state of DPP3, suitable for future *in vivo* applications to investigate the physiological role of DPP3 and its involvement in pathophysiological conditions.

## Introduction

Human dipeptidyl peptidase 3 (DPP3, EC 3.4.14.4) is a zinc-dependent dipeptidyl peptidase with a molecular mass of about 82.6 kDa. It is mainly a cytosolic enzyme, although membrane associated forms have been reported [1–4]. The enzyme is ubiquitously expressed and is evolutionarily conserved among prokaryotes and eukaryotes [5]. DPP3 belongs to the M49 family of aminopeptidases that is characterized by the presence of the conserved HEXXGH and EEXRAE(D) motifs, which are involved in the coordination of Zn^2+^ cofactor in the active site [6]. DPP3 sequentially cleaves dipeptides (e.g. Arg-Arg-, Ala-Arg-, Asp-Arg- or Leu-Arg-) from the free N-terminus of several bioactive peptide-substrates with the most prominent substrate being Angiotensin II, the main effector of the renin-angiotensin system (RAS) [7,8]. Despite the first identification of DPP3 in bovine pituitary gland more than 50 years ago [9], the exact physiological role of intracellular DPP3 remains unclear since its known substrates are extracellular bioactive peptides.

An involvement of DPP3 in blood pressure regulation, inflammatory processes and pain modulation was proposed [5] due to DPP3’s high *in vitro* affinity to opioid peptides like enkephalins [5], endomorphins [10] and angiotensins (Ang II, Ang III and Ang IV) [11]. In fact, Pang et al. showed antihypertensive effects of recombinant rat DPP3 injection in Ang II-infused mice, suggesting DPP3 could be employed as a therapeutic agent for hypertension [12]. Furthermore, DPP3 levels are increased in certain types of cancer, such as lung cell carcinoma [13], breast [14] and ovarian cancer [15–17]. Recently, Rehfeld et al. introduced methods for the specific detection of DPP3 protein concentration and activity in human plasma samples. Using these immunoassays the authors showed an increase of plasma DPP3 levels in severely ill septic patients. Furthermore, high DPP3 plasma levels were associated with disease-severity and high mortality risk [18]. Its potential involvement in several pathological conditions makes DPP3 an interesting target for further *in vivo* and *in vitro* studies to determine its exact biological function and pathophysiological roles.

In the past, DPP3 was purified from several mammalian biological samples such as rat brain, porcine skeletal muscle, bovine pituitary and human erythrocyte lysates [8,9,19,20]. Although all published preparations led to a purified DPP3 product, they all differ in purity and specific activity, making it difficult to assess DPP3’s physiological role. DPP3 was additionally expressed recombinantly in bacteria and insect cells and purified as an affinity-tagged protein [5]. Recombinant proteins may lack post-translational modifications that impact their biochemical properties [21]. Furthermore, affinity tags can influence protein activity and/or increase protein solubility even when the target is misfolded, leading to high purification yields, but poor active protein recovery [22]. Especially, recombinant expression in bacteria leads to high endotoxin content that requires additional purification steps [23].

To overcome these limitations, we established a simple, but highly effective purification method of native DPP3 from human blood cell lysates (BCL). Cruor from blood sample collections is broadly available and relatively low-priced. Therefore, the usage of a blood cell lysate is a cost- and resource-effective biological source for native DPP3. Combined with DPP3-specific activity and concentration assays [18], we could show that our method results in high yields of pure native DPP3 without specific activity loss during the purification procedure or during protein storage. The resulting native product can be used for future studies to dissect human DPP3 function *in vivo* and *in vitro*.

## Materials and methods

### Preparation of cleared blood cell lysate (BCL)

Human blood cell lysate (BCL), hemolysed by ultrasonication, were purchased from InVent Diagnostica GmbH, Hennigsdorf, Germany.

### Purification of DPP3

Two liters of BCL were pre-cleared with 100 ml Sepharose 4B (S4B) resin (Sigma-Aldrich), to reduce unspecific binding of lipoproteins and lipids to resins used in future purifications steps. The flow-through was collected. The S4B-resin was washed with a total of 370 mL PBS buffer, pH 7.4, and the wash fraction was combined with the collected flow-through, resulting in a total volume of 2370 mL. The resin was discarded.

The second purification step is an immuno-affinity chromatography using a specific monoclonal anti-DPP3 antibody (AK2552, produced as described for other anti-DPP3 antibodies by Rehfeld et al. [18]), followed by an ion exchange chromatography step.

For the immuno-affinity chromatography, 110 mg of AK2552 were coupled to 25.5 mL of UltraLink Hydrazide Resin (Thermo Fisher Scientific) according to the manufacturer’s protocol. The coupling efficiency was 98% determined by quantification of uncoupled antibody via Bradford. The resin-antibody conjugate was equilibrated with wash-binding buffer (PBS, 0.1% TritonX-100, pH 7.4), combined with cleared BCL and incubated at 4°C under continuous stirring for 2h. The incubation mixture was equally divided in 10 parts and filled in polypropylene columns. The columns were centrifuged at 1000xg for 30 seconds resulting in 2.5 mL of DPP3-loaded resin per column. The flow-through was discarded. Each column was then washed 5 times with 10 mL of wash-binding buffer by gravity-flow. DPP3 was eluted by placing each column in a 50-mL falcon tube containing 2 mL of neutralization buffer (1M Tris-HCl, pH 8.5), followed by addition of 10 mL of elution buffer (100 mM Glycine-HCl, 0.1% TritonX-100, pH 3.5) per column and immediate centrifugation for 30 seconds at 1000xg. The pH of the neutralized eluates was 8.0. The elution step was repeated 5 times, presence of DPP3 was identified using the DPP3-LIA (DPP3-Luminescent Immuno Assay) [18] and respective fractions were combined.

The combined eluates (360 mL) were loaded on a 5 mL HiTrap Q-sepharose HP column (GE Healthcare) equilibrated with IEX-buffer A1 (100 mM Glycine, 150 mM Tris, pH 8.0; Äkta Start, GE Healthcare). After sample loading, the column was washed with five column volumes of IEX Buffer A2 (12 mM NaH_2_PO_4_, pH 7.4). Elution of DPP3 was achieved by applying a sodium chloride gradient over 10 column volumes in a range of 0–1 M NaCl using IEX-buffer B (12 mM NaH_2_PO_4_, 1 M NaCl, pH 7.4). The eluates were collected in 2 mL fractions. Fractions showing highest activity (DPP3-ECA) were pooled and stored in 12mM NaH_2_PO_4_, 400 mM NaCl, pH 7.4 at -20°C until usage.

### Endotoxin load determination

Endotoxin load determination was carried out by InVivo Biotech Services GmbH, Hennigsdorf, Germany using an Endosafe PTS LAL testing platform.

### Analytical size exclusion chromatography

The analytical size exclusion chromatography (SEC) was performed using the Knauer HPLC Smartline system in combination with a Shodex KW-803 silica-gel size exclusion column. The running buffer was PBS, pH 6.8, applied with a flow-rate of 0.5 mL/min. Presence of proteins was monitored via absorption at 280 nm during the run.

### Protein purity analysis and Western Blotting

Protein purity estimation was carried out using SDS-PAGE on 4–20% precast acrylamide gels (Biorad 4–20% Mini-Protean TGX Precast Protein Gels, 15-well). Gels were stained with Coomassie Brilliant Blue R-250 Staining solution (Biorad).

Transferring proteins from SDS-gels to PVDF membranes (BioRad) was performed using the BioRad Turbo blotting system (Trans-Blot Turbo Transfer System). After the transfer, membranes were washed three times with PBS-T (PBS, 0.08% Tween-20, pH 7.4) and blocked with 5% BSA in PBS-T. Afterwards, membranes were incubated with the primary anti-DPP3 mAb (murine AK1967, 2 μg/mL in PBS-T with 1% BSA) over night at 4°C on a shacking platform and washed five times. Membranes were incubated with secondary antibody (Anti-mouse IgG, HRP-linked antibody, Cell Signaling Technology, 1:5000 in PBS-T with 1% BSA) at room temperature for 1 hour on a rocking shaker. Afterwards membranes were washed three times. Membranes were developed by incubation with ECL Prime Western Blotting Detection Reagents (Amersham GE Healthcare). Chemiluminescence was detected using the LAS 500 imager system (GE Healthcare).

### Protein determination

Total protein amount was estimated using the Micro Lowry Protein Kit [24] (Sigma Aldrich) according to manufacturer’s manual with slight modifications. Briefly, the volume in sample preparation was scaled-down by a factor of five. 150 μL of sample were used for measurement in 96-well format using a plate reader (Molecular Devices, Spectra MAX 250). Samples were measured in duplicates at 600 nm. Standards, blanks and samples were treated equally.

DPP3 concentration was determined via a DPP3-LIA as previously described [18], while the incubation time was reduced to 1h. Samples and calibrators were measured in duplicates.

### Activity determinations

The activity of DPP3 was determined using either a soluble activity assay (SAA) [8,9,25] or an enzyme capture activity assay (ECA) as previously described [18], while the incubation time for capturing DPP3 was reduced to 1h. Samples and calibrators were measured in duplicates.

SAA was carried out as previously described [8,9,25]. In short: DPP3-dilutions, 10 μL per well (in 50 mM Tris-HCl, 0.1 mM CoCl_2_, pH 8.6), were added into black non-binding polystyrene microtiter plates (Greiner Bio-One). Subsequently, 90 μL of reaction buffer (50 mM Tris-HCl, 0.1 mM CoCl_2_, 45 μM Arg_2_-ß-naphtylamide (Arg_2_-ßNA), pH 8.6) were added and plates were incubated for 1h at 37°C and atmospheric pressure. The initial fluorescence at t = 0 min and the fluorescence of the product ß-naphtylamine (ß-NA) at t = 1h were measured using the Twinkle LB 970 fluorometer (Berthold Technologies GmbH, excitation at 340 nm, emission detected at 410 nm). Arg-ß-naphtylamide (Arg-ßNA) or Leu-ß-naphtylamide (Leu-ßNA) hydrolyzing activities were assayed under same conditions using reaction buffers with pH 7.5 or 6.5. The activity of DPP3 was determined using an 8-point calibration curve of ß-NA (0.05–100 μM). Samples and calibrators were measured in duplicates. Activities are expressed in U (μmol product formed per minute).

### Determination of optimal IAP elution buffer

DPP3-containing BCL (50 mL) was incubated with 1.8 mL of AK2552-coupled UltraLink Hydrazide Resin (1.42 mg AK2552 per mL of resin, Thermo Fisher Scientific) in a polypropylene column for 90 minutes upon continuous rotation. The flow-through was collected by gravity-flow. Afterwards the resin was washed five times with 10 mL of wash-binding buffer and resuspended in 1.8 ml of wash-binding buffer. Three polypropylene columns were filled with 0.7 mL each of DPP3-loaded resin-slurry with subsequent centrifugation at 1000xg resulting in 0.35 mL of resin per column. The flow-through was discarded. DPP3 was eluted by placing each column in 15-mL falcon tubes containing 0.7 mL of neutralization buffer (1M Tris-HCl, pH 8.5), followed by addition of 3.5 mL of elution buffer (100 mM Glycine-HCl, 0.1% TritonX-100 pH 3, pH 3.5 or pH 4) per column and immediate centrifugation for 30 seconds at 1000xg. The elution step was performed once. Starting material, flow-through and eluates were tested using DPP3-LIA for presence of DPP3. Eluted DPP3 was further purified via IEX as described above and used for DPP3 pH-stability investigations.

### pH dependence of DPP3 stability

For assaying the pH dependence of DPP3 stability, native DPP3 was diluted in buffers of different pH (Table 1) to a concentration of 73.4 μg/mL in a total volume of 200 μL and incubated at room temperature. At different incubation time points (30 sec, 5 min and 1 h), 8 μL of each incubation mixture were neutralized by dilution in 500 μL ECA-assay-buffer [18] and stored until measurement at 4°C for maximum 60 minutes. DPP3 activity was determined via the DPP3-ECA.

**Table 1:** Buffers used for determination of pH stability of human DPP3.

pH	Buffer
2, 3, 3.5, 4	100 mM Glycine-HCl
4, 5, 6	100 mM Citrate
6, 7, 8	57.8 mM Na_2_HPO_4_, 42.2 mM NaH_2_PO_4_
8, 9, 10	100 mM Glycine-NaOH

### Protein stability in storage buffer

Freshly purified native DPP3 enzyme was diluted in storage buffer (12mM NaH_2_PO_4_, 400 mM NaCl, pH 7.4) in presence or absence of 10% glycerol to a concentration of 200 μg/mL, sterile filtered, divided in several aliquots and frozen at -20°C. Every 24h aliquots of DPP3 were thawed and stored at room temperature or 4°C until measurement of DPP3 activity. To asses freeze-thaw stability, aliquots of DPP3 were repeatedly thawed and subsequently frozen at -20°C every 24h. DPP3 activity was determined via DPP3-ECA after 7 days of storage or after 5 freeze-thaw cycles. Protein that did not undergo several freeze-thaw cycles or storage at 4°C or room temperature was set to have 100% activity in DPP3-ECA. Determination of melting temperature of DPP3 in sterile storage buffer in presence or absence of 10% glycerol or in sterile phosphate buffer saline (PBS) was determined using differential scanning fluorimetry (DSF) applying the sypro orange reagent (Thermo Fisher Scientific) as previously described [26]. Concentration of DPP3 in DSF measurements was 0.1 μg/mL.

### Mass spectrometric identification and molecular weight determination of DPP3

For protein identity, purified native DPP3 (7 μg) was applied on a 10%-SDS Gel under reducing conditions. The single band migrating between 100 and 70 kDa was cut under sterile conditions and stored at room temperature until analysis. Mass spectrometric analysis via MALDI-TOF MS was carried out by Proteome Factory AG, Germany.

For exact molecular weight determination via HPLC-ESI-MS, purified DPP3 (1.5 mg/mL) was diluted ten-fold with water with subsequent addition of tris-2-carboxyethyl-phosphine (TCEP) to a final concentration of 25 mM. The sample was incubated at 37°C for 30 min. An aliquot with 1.8 pmol DPP3 was applied on a HPLC-assisted reversed phase column (Zorbax 300SB-C8, 0.3x100 mm, 3.5 μm, Agilent) and a gradient elution with 0.1% formic acid in water (solvent A) and 0.1% formic acid in acetonitrile (solvent B) was performed (flow rate: 8μl/min). The column effluent was directed to an Orbitrap Velos (Thermo Fisher Scientific) using an HESI-II electrospray interface (Thermo Fisher Scientific). Charge state deconvolution was performed using the ZNova algorithm (Novatia LLC, Newtown, PA, USA). Mass spectrometric analysis via HPLC-ESI-MS was carried out by Proteome Factory AG, Germany.

## Results

A three-step purification protocol for native human DPP3 from BCL was established comprising a preclearance step with Sepharose 4B and an immuno-affinity purification (IAP) followed by an ion-exchange purification. Purification status and activities were monitored during the whole process using the DPP3-LIA and DPP3-ECA (Table 2). Both assays have the advantage of capturing the enzyme, which allows a washing step to remove undesired material, such as hemoglobin and other interfering components of blood cell lysate [25]. We also employed the soluble activity assay [8], which was used by other groups for DPP3 activity determination so far, to allow a balanced comparison between our purification method and others described in the literature.

**Table 2:** Purification of DPP3 from human BCL.

Step	Sample volume in mL	DPP3 amount in % (LIA)^a)^	Total protein in mg	Total activity in μmol/min (ßNA-ECA)	Specific activity in ECA-U/mg^b)^	Purification factor^c)^	Yield in %^d)^
BCL	2000	100	213733	57	0,00027	-	100
S4B	2370	91	204160	55	0.00027	1	97
IAP	360	73	71.2	46.1	0.65	2407	81
IEX	4	68	6.6	38.7	5.9	21852	68

^a)^ Amount of DPP3 was determined via DPP3-LIA and is expressed relative to the starting material that was set as 100%.

^b)^ Specific ECA-activity was calculated from total DPP3-ECA activity and the total protein content. Units are defined as μmol product formed per minute in ECA

^c)^ The purification factor is the quotient of specific ECA-activity after and before each purification step.

^d)^ The yield represents the recovery of total DPP3-ECA activity after each purification step relative to the starting material

### Determination of DPP3 pH stability

**Fig 1:**
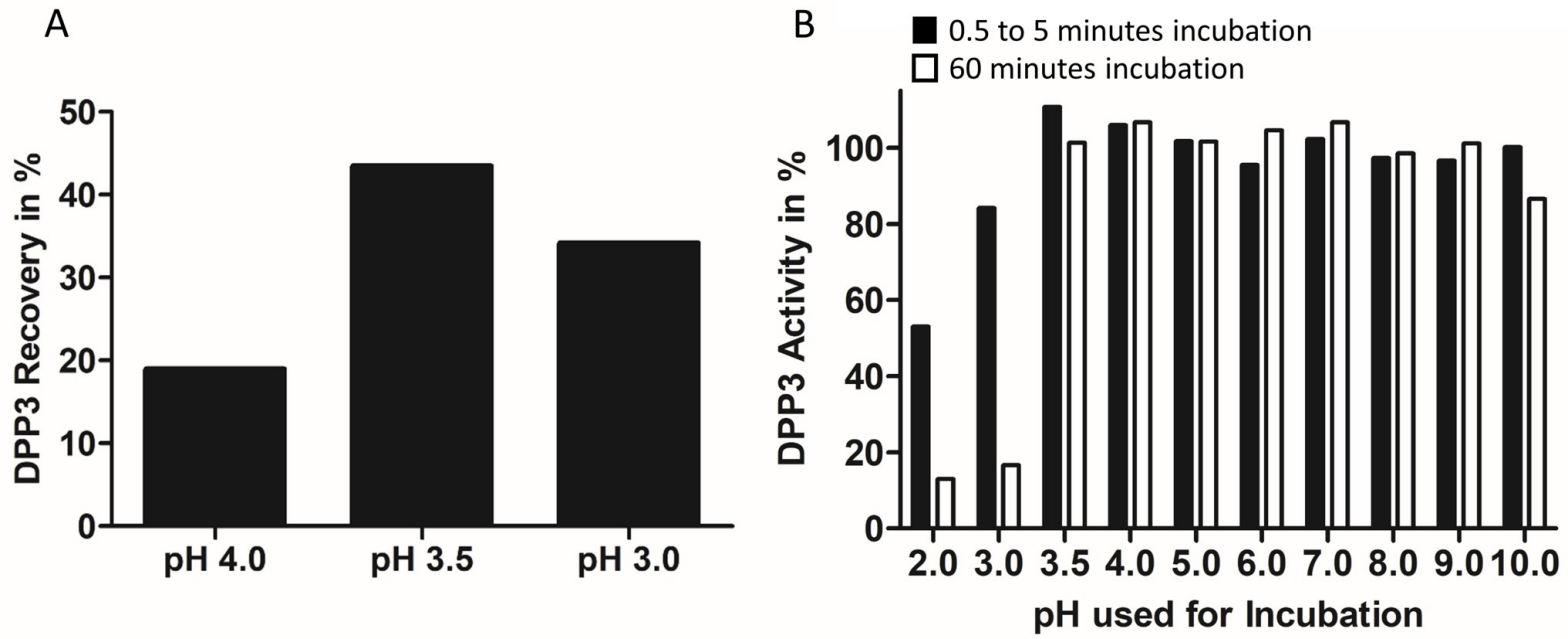
pH Stability of DPP3 and elution efficiency of native DPP3 at specific pH. **(A)** One-step elution of native DPP3 during IAP using buffers of different pH values shown in the figure. After elution, presence of DPP3 was measured via DPP3-LIA. DPP3 amount captured by the resin (Amount of DPP3 in starting material (BCL) substracted by amount of DPP3 in flow-through) was set as 100%. (B) DPP3 was incubated in buffers of different pH values as indicated in the figure. Aliquots of the incubation mixture were neutralized either after 30 seconds (pH 2.0–4.0) or after 5 minutes (pH 5.0 –pH 10.0) (black bars) and after 60 minutes of incubation (white bars). DPP3 activities were measured in DPP3-ECA. The activity of an untreated control (DPP3 in storage buffer, pH 7.4) was set as 100%.

Elution of antibody-bound antigens during IAP requires acidic buffer conditions. To investigate, whether captured DPP3 may be eluted during IAP using a buffer in the range of pH 3–4, we compared elution-efficiency of buffers with pH 3, 3.5 and 4. Among the three buffers, the buffers with pH 3 and 3.5 showed the highest DPP3 recovery determined with DPP3-LIA after one elution step (Fig 1A). Since DPP3 activity and stability was reported in the past to be affected by low pH [8,19], we tested the behavior of native DPP3 under several pH conditions including pH 3.0 and pH 3.5 (Table 1). The Arg_2_-ßNA hydrolyzing activity remained stable for at least 1 hour upon incubation of DPP3 from pH 3.5 to pH 9 (Fig 1B). On the other hand, we observed a rapid inactivation at pH 2 and 3 with a loss of more than ~80% of the initial activity within 1 hour (Fig 1B). Therefore, we used a buffer-system with a pH of 3.5 to elute antibody-bound DPP3 during IAP, since it represents the most reasonable compromise between DPP3 recovery and remaining activity (Fig 1A and 1B).

### Immuno-affinity purification (IAP)

**Fig 2:**
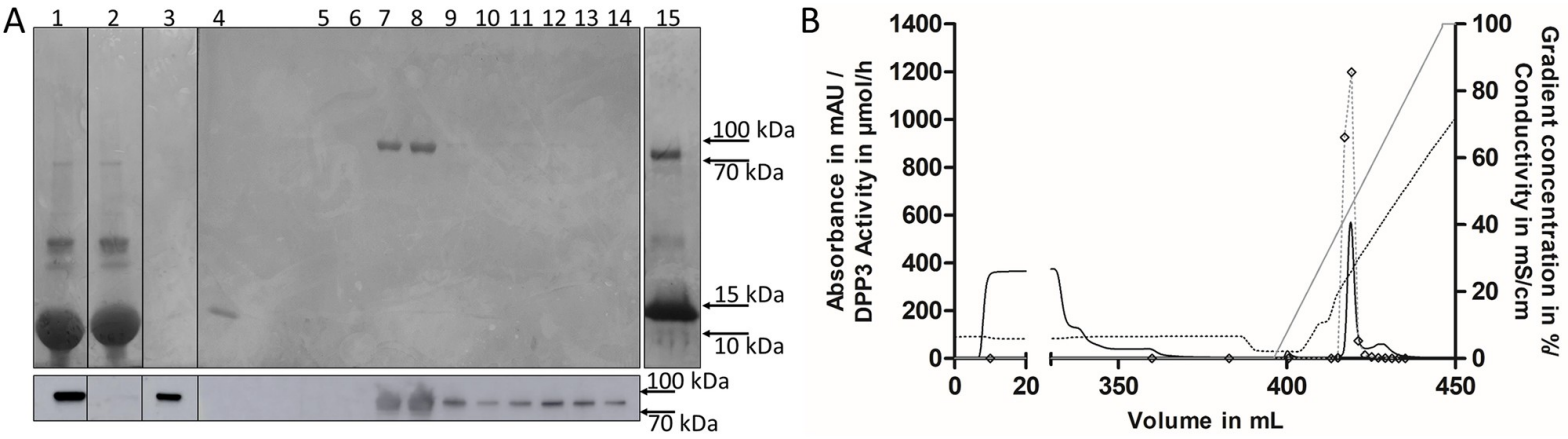
Coomassie staining of DPP3 purification fractions and chromatogram of DPP3-IEX purification. **(A)** Top: Coomassie staining of purification fractions on a 4–20% gradient gel. Bottom: Western Blot of purification fractions shown in A-top. Lane 1: Starting material in 1:10 dilution, Lane 2: IAP flow-through in 1:10 dilution, Lane 3: Combined IAP eluates in 1:10 dilution, Lane 4: Concentrated IEX flow-through in 1:20 dilution, Lanes 5–14: Fractions 12–21 from the IEX run in 1:20 dilution. Lanes 7 and 8 contain the main DPP3 fractions contributing to the elution peak in Fig 2B, Lane 15: undiluted and two-fold concentrated combined IAP eluates shown in lane 3. Protein ladder used: Spectra Multicolor Broad Range Protein Ladder (Thermo Fisher Scientific). (B) DPP3 containing fraction from the immuno-affinity purification (360 mL) were loaded on the Q-sepharose resin using the Äkta Start sample pump (0–370 mL). After loading the column was washed with 5 CV of IEX-buffer 2 (370–395 mL). Elution of DPP3 was started by application of a NaCl gradient over a volume of 10 CV (395–445 mL). Activity in elution fractions was determined via DPP3-ECA. Solid gray line: salt gradient in %, solid black line: absorbance at 280 nm in mAU, dotted gray line with squares: Arg_2_-ßNA hydrolyzing activity in μmol/h, dotted black line: conductivity in mS/cm.

The main step of DPP3 purification included the enrichment of DPP3 from pre-cleared BCL using an immuno-affinity purification (IAP) step with an immobilized specific monoclonal anti-DPP3 antibody (AK2552). We performed the elution of DPP3 captured by the immobilized antibody using an acidic buffer at pH 3.5 in 5 steps. DPP3 containing eluates (1–3) were combined and further purified in ion-exchange chromatography. DPP3-LIA and -ECA analysis showed that the IAP purification step rescued approximately 80% of the total DPP3 activity when compared to the starting material, resulting in a purification factor of 2,407 (Table 2). While the IAP purification step reduced the total protein content of the sample from 204,160 mg to 71 mg (Table 2), hemoglobin remained as the main contaminant in the combined IAP eluates, as shown by SDS-PAGE analysis (Fig 2A, lanes 3 and 15).

### Ion-exchange purification

To remove hemoglobin and other contaminants, we implemented an additional ion-exchange purification step by using a Q-sepharose column. Since the experimentally determined pI of DPP3 is 4.5–4.6 [8] and the combined IAP eluates had a pH of 8, additional pH adjustment was not required to ensure binding of DPP3 to the positively charged Q-sepharose resin. After loading of DPP3 sample on the Q-sepharose resin, a large portion of hemoglobin was detected by SDS-PAGE in the flow-through. DPP3 was not detected in the flow-through, neither with Coomassie brilliant blue staining (Fig 2A, top-lane 4), Western Blot (Fig 2A, bottom-lane 4) nor with the DPP3-LIA assay. The elution of DPP3 from the Q-sepharose resin was achieved by applying a salt gradient in the range of 0–1 M NaCl. DPP3 eluted as a sharp single peak at a salt concentration range between 400–500 mM (Fig 2B). We verified the presence of DPP3 in the corresponding fractions via SDS-PAGE and DPP3-ECA (Fig 2A and 2B). DPP3 in fractions eluting at higher salt concentrations accounted for 3% of total recovered DPP3 activity and was discarded. The fractions containing the highest activity showed the presence of a single protein band in SDS-PAGE migrating between 70 and 100 kDa, as expected for DPP3 (83 kDa), without any detectable impurities in Coomassie-stained gels (Fig 2A, lanes 7 and 8). The presence of DPP3 in the pooled fractions was additionally verified via mass spectrometry (sequence coverage: 80%, Score: 3421). Due to the additional ion exchange purification step, we reduced the total protein content to 6.6 mg representing approximately 68% of the total DPP3 amount and total DPP3 enzyme activity when compared to the starting material.

### Characterization of DPP3 preparation

**Fig 3:**
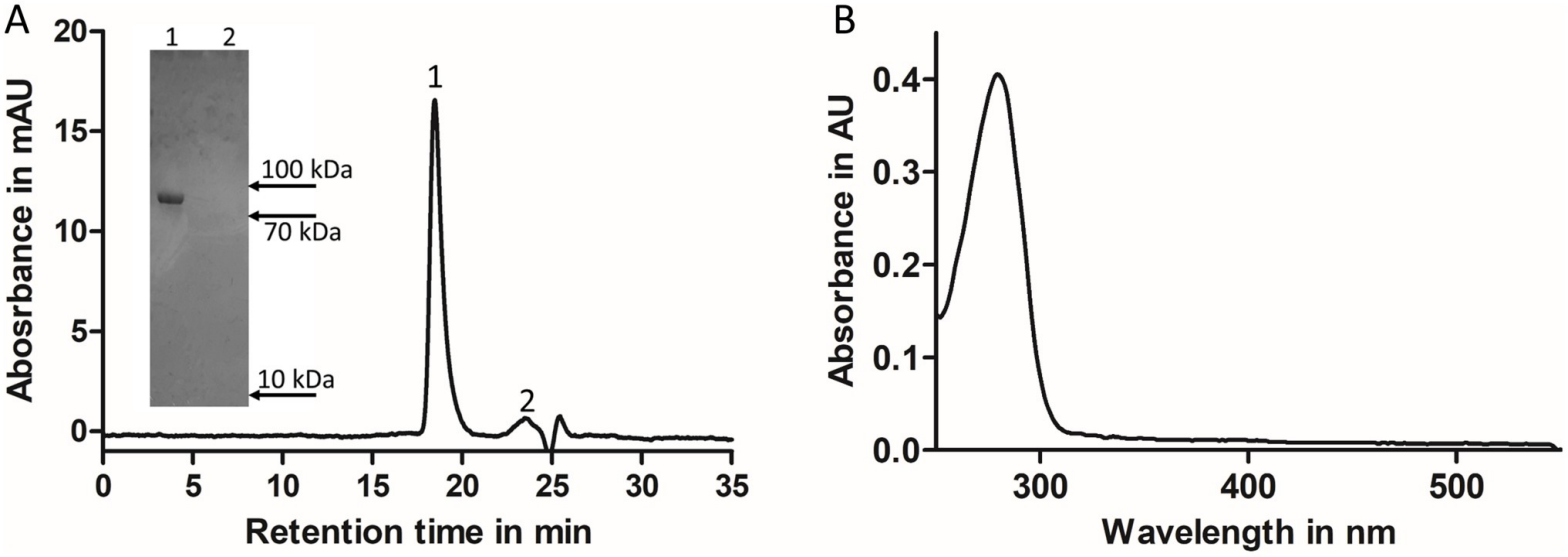
Analytical size exclusion and UV-vis spectrum of purified native DPP3. **(A)** Analytical Size Exclusion Chromatogram of native hDPP3. DPP3 purified via IAP and IEX was applied on a Shodex silica gel-size exclusion column with a flowrate of 0.5 mL/min. Fractions belonging to peaks 1 and 2 were collected and analyzed via SDS-PAGE (inlay). (B) UV-vis spectrum of purified native DPP3 in the range of 250-550nm. The spectrum was recorded in storage buffer at room temperature using DPP3 in a concentration of 324 μg/mL.

To address the purity of the DPP3 preparation, we performed an HPLC-based analytical size exclusion chromatography. The resulting elution profile (Fig 3A) showed one major peak containing pure DPP3 (Fig 3A, inlay). The areas under peaks 1 and 2 (Fig 3A) imply a purity of our DPP3 preparation being ≥ 95%. Thus, in our final preparation containing 6.6 mg of total protein, DPP3 accounts for approximately 6.3 mg of the protein amount. The ultraviolet-visible (UV-vis) spectrum of purified native DPP3 shows one single protein-absorption band at 280 nm (Fig 3B). No contaminating proteins with chromophoric groups, e.g. hemoglobin, were detected in the UV-vis spectroscopy. Photometric determination of DPP3 concentration in our preparation (ɛ_280_ = 1.225 mL*mg^-1^*cm^-1^) revealed a total DPP3 content of 5.8 mg supporting the SEC-determined purity of our DPP3 preparation. In addition, an endotoxin determination test showed our preparation to be free of endotoxins (below 3 EU/mg).

**Fig 4:**
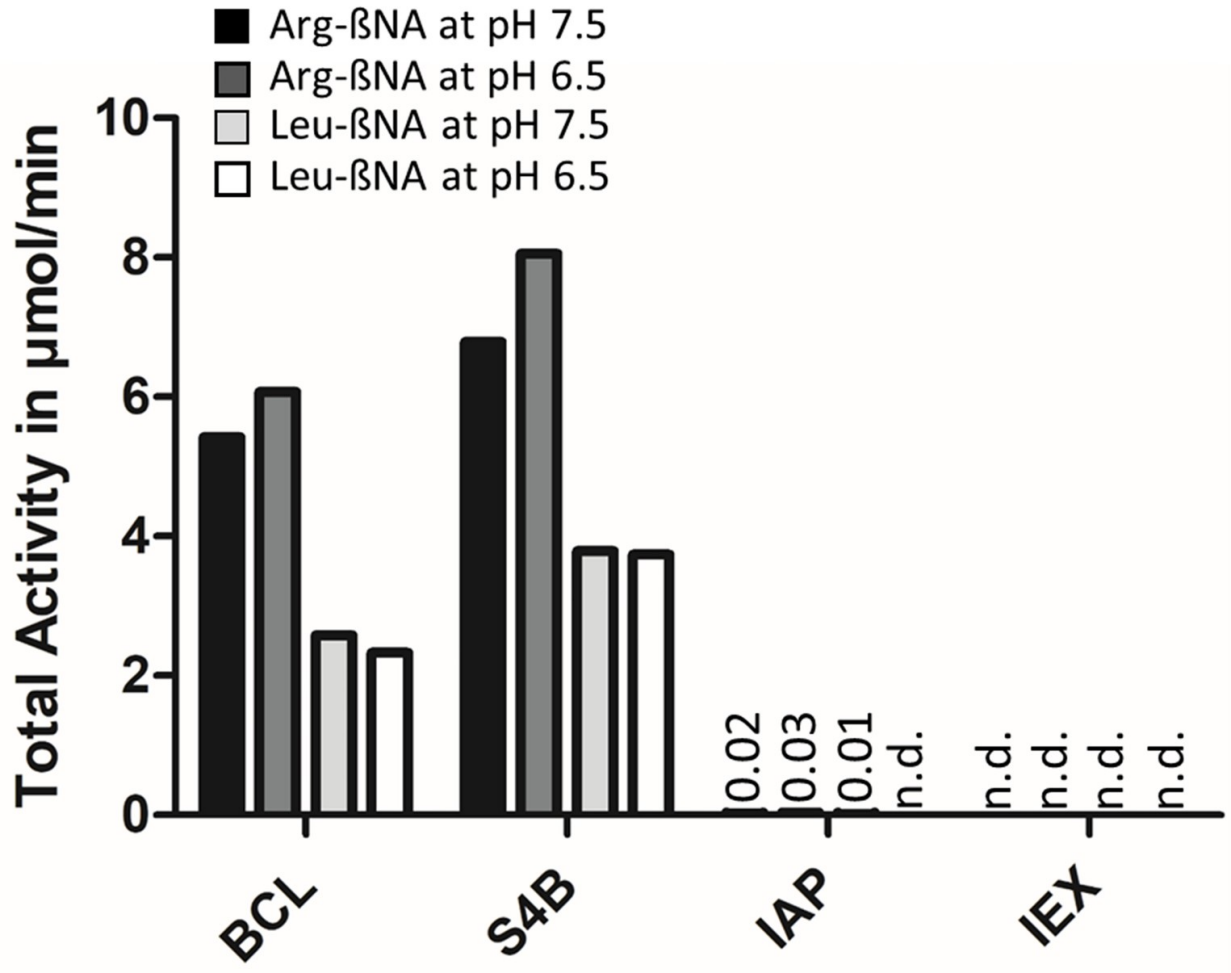
Arg-ßNA and Leu-ßNA hydrolyzing activities of DPP3 containing fractions during purification. DPP3 activity was assayed at pH 7.5 and pH 6.5 with Arg-ßNA or Leu-ßNA as substrates. All tested fractions were diluted to a DPP3 content of 100 μg/mL (DPP3-LIA) and assayed as described in materials and methods. Black bar: reaction at pH 7.5 with Arg-ßNA as substrate, dark-grey bar: reaction at pH 6.5 with Arg-ßNA as substrate, light-grey bar: reaction at pH 7.5 with Leu-ßNA as substrate, white bar: reaction at pH 6.5 with Leu-ßNA as substrate. BCL: Blood cell lysate, S4B: Sepharose 4B, IAP: immuno-affinity purification, IEX: ion-exchange.

The activity of this highly pure product determined in a soluble activity assay [8] with Arg_2_-ßNA was 49.1 ± 1.6 U/mg (k_cat_ = 67.5 s^-1^) at pH 8.6 and 22.2 ± 1.6 U/mg (k_cat_ = 30.5 s^-1^) at pH 7.5 calculated using the actual DPP3 amount in the preparation. The purified protein showed no activity when Arg-ßNA or Leu-ßNA were used as substrates (Fig 4), indicating the absence of contaminating aminopeptidases releasing Arg- or Leu-residues. Furthermore, the data in Fig 4 implies that the immuno-affinity step recovers specifically DPP3, since the remaining Arg-ßNA and Leu-ßNA hydrolyzing activity was below 0.5% when compared to the Sepharose 4B precleared BCL.

**Fig 5:**
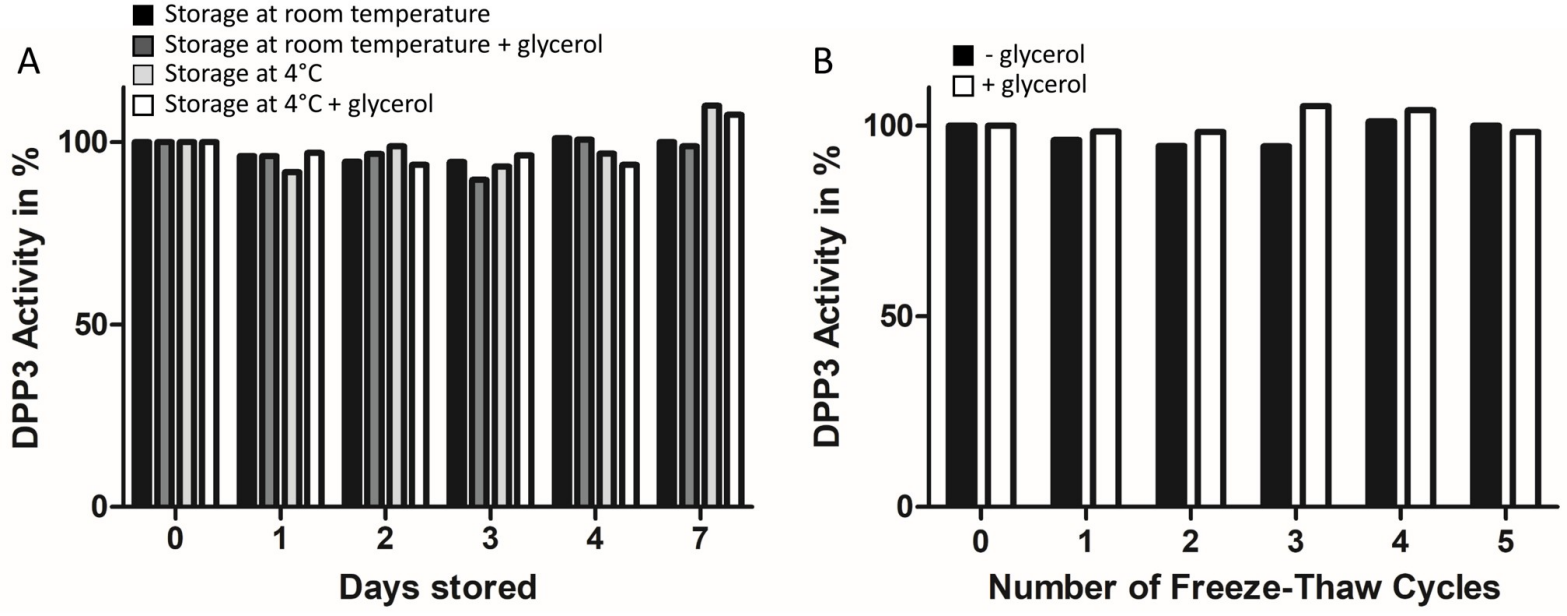
Freeze-thaw stability and storage stability of native hDPP3. **(A)** Purified and sterile filtered DPP3 (200 μg/mL) in storage buffer in presence or absence of 10% glycerol was stored for 1 to 7 days at 4°C or room temperature. Activity of stored DPP3 was determined via DPP3-ECA. Activity of DPP3 stored for a period of 7 days at -20°C was set as 100%. Black bar: DPP3 stored at room temperature in phosphate buffer, dark-grey bar: DPP3 stored at room temperature in presence of glycerol, light-grey bar: DPP3 stored at 4°C in phosphate buffer, white bar: DPP3 stored at 4°C in presence of glycerol. (B) Purified and sterile filtered DPP3 in 12mM NaH_2_PO_4_, 400 mM NaCl, pH 7.4 (black bar) or in 12mM NaH_2_PO_4_, 400 mM NaCl, 10% glycerol (white bar), pH 7.4 was applied to 1 freeze-thaw cycles in 24h. All aliquots were stored at -20°C until activity determination via DPP3-ECA. Activity of protein stored at -20°C for 5 days (0 freeze-thaw cycles) was set as 100%.

The purified DPP3 enzyme showed no loss of activity measured by the DPP3-ECA assay over a period of 7 days, when stored in storage buffer at -20°C, 4°C or room temperature (Fig 5A). In addition, we observed no loss of activity after 5 freeze-thaw cycles of purified DPP3 in storage buffer (Fig 5B). The absence of glycerol in the storage buffer had no impact on storage- or freeze-thaw stability of native DPP3 enzyme (Fig 5B). Furthermore, DSF revealed a melting temperature of DPP3 being 55–56°C in storage buffer with or without glycerol, as well as in PBS buffer, allowing usage of native DPP3 in *in vivo* applications.

Finally, HPLC-ESI-MS analysis of the purified DPP3 enzyme revealed a single species in our preparation having a molecular weight of 82487 Da (S1 Fig), which is consistent with the molecular mass reported by Abramic et al. (82500 Da) [11].

## Discussion

The here described procedure allows a fast and highly efficient purification of DPP3 from human BCL resulting in a 22,000-fold purification, providing a yield of 68% and a purity of ≥ 95% within three purification steps. The relative amount of purified DPP3 determined via DPP3-LIA correlates well with the rescued DPP3 activity, measured by DPP3-ECA, throughout the whole purification, indicating no loss of specific DPP3-activity during the whole process.

The purification step that mainly contributed to the reported purification factor and yield is the immuno-affinity purification. To reach DPP3 purification yields described in this work, the antibody used for immuno-affinity purification had to fulfill two main criteria. On the one hand, the used antibody needs to ensure efficient capturing of DPP3 from a complex starting material such as human blood cell lysate. On the other hand, the chosen antibody needs to assure efficient elution of captured DPP3 at pH conditions that are non-critical to the protein activity. As shown in our study (Fig 1B), DPP3 is sensitive to low-pH conditions, which lead to inactivation of the protein. The IAP-antibody used in this study (AK2552) accomplishes both: it captures the majority of the DPP3 from blood cell lysate (Table 2) and enables the use of elution conditions at pH values that preserve DPP3 activity resulting in 81% DPP3 recovery (Fig 1A, Table 2). A direct comparison of several in-house anti-DPP3 antibodies, including those previously described by Rehfeld et al. (AK2553 and AK2555) [18] revealed that the two described criteria were fulfilled only by AK2552.

Due to the exceptional stability of antibodies even under harsh conditions, the used antibody-resin conjugate can be reused several times without capacity loss. A requirement for the affinity resin recycling is the clearance of the BCL from unspecific-binding proteins and lipids achieved via Sepharose 4B. Since hemoglobin was unspecifically co-purified as the main contaminant during IAP, an additional purification step via ion-exchange was necessary, which led to a highly pure and stable DPP3 product.

The highest DPP3 purification yield from mammalian tissues reported to date was 49% for DPP3 from porcine spleen [27], and reached purification factors were in the range of 300 and 12,000 [9,19,20,27–29]. As an exception, Abramic and coworkers described a 40,000-fold purification factor with a yield of 35% for human erythrocyte DPP3 and a specific activity of 21 U/mg after 4 isolation steps [8]. When assayed under same conditions, our native hDPP3 preparation shows more than two-fold higher specific activity of 49.1 U/mg suggesting higher purity in comparison to DPP3 purified by Abramic and coworkers. However, since Abramic et al. measured DPP3 activity using the soluble activity assay (SAA), one main aspect needs to be considered with respect to the purification factor described by Abramic et al. and this work. For the description of our purification procedure, we used the highly specific DPP3-ECA that eliminates common disturbing factors (hemoglobin and contaminating peptidases) when DPP3 activity is measured via the Arg_2_-ßNA substrate [18,25]. In contrast, the SAA is negatively affected by hemoglobin and its ability to quench the ßNA-emitted fluorescence signal [25]. Thus, application of the SAA will result in an underestimation of DPP3 activity in hemoglobin rich starting material. This will probably lead to an overestimated high total purification factor, when the activity of the final, purified DPP3 preparation is compared to the one obtained from the hemoglobin-contaminated starting-material, as described by Abramic et al. The use of DPP3-ECA represents a more accurate way of determining DPP3 activity during purification, especially when a comparison to hemoglobin-containing starting material needs to be done. Since the total amount of DPP3 immobilized by solid phase in ECA cannot be absolutely determined, the specific ECA-activity shown in Table 2 cannot be directly compared to the specific activity determined in SAA using the highly pure DPP3 preparation.

The molecular weight of our DPP3-preparation determined via HPLC-ESI-MS was 82487 Da, which is in good agreement with the previously reported value of 82500 Da [11] and with the theoretical molecular mass of human DPP3 (82589 Da, Uniprot Entry Q9NY33). The actual molecular weight of 82478 Da implies cleavage of N-terminal methionine in position 1 and posttranslational N-terminal acetylation of alanine in position 2. The absence of further species in HPLC-ESI-MS (S1 Fig) in combination with the absence of Arg-ßNA and Leu-ßNA hydrolyzing activities in the final preparation as well as the high specific activity in SAA with Arg_2_-NA as substrate support the determined purity of ≥ 95%.

The highly pure human DPP3 obtained via this purification procedure can be used for further investigations of the DPP3 role in the onset of pathological conditions in animal studies, by inducing high DPP3 plasma levels as observed in critically ill patients [18]. Pang et al. used recombinant rat DPP3, produced as a GST-tagged construct in bacteria, to treat hypertensive rats as a function of Angiotensin II degradation [12]. Unfortunately, no activity with Arg_2_-ßNA as substrate were reported by Pang et al., which makes a direct comparison to our preparation not possible. Nevertheless, human DPP3 shows higher Arg_2_-ßNA hydrolyzing activities at physiological pH when compared to rat DPP3 (k_cat_ = 18.12 and 6.9, respectively) [11]. On the other hand, higher angiotensin III-binding affinity was reported for rat DPP3 (*K*_*i*_^hDPP3^ = 0.088 μM, *K*_*i*_^ratDPP3^ = 0.024 μM) [11], while binding of angiotensin II was comparable for both, human and rat DPP3 (*K*_*i*_^hDPP3^ = 0.83 μM, *K*_*i*_^ratDPP3^ = 0.66 μM) [11]. The described comparison of rat and human DPP3 was performed in the presence of 10 μM Co^2+^, since rat DPP3 is inhibited at Co^2+^ concentrations greater than 20 μM [11]. Human DPP3 is not inhibited at higher Co^2+^ concentrations and might show greater differences to rat DPP3 when assayed with 100 μM of Co^2+^. In summary, rat and human DPP3 appear not to be identical catalysts under physiological pH. In this context, an intriguing question is whether high hDPP3 levels in animal studies will lead to comparable results as described by Pang et al. [12]. Thus, the native, highly pure and endotoxin-free DPP3 obtained by us with the described purification procedure could be used for in-depth studies of DPP3 physiological function.

Finally, the here obtained purified native human DPP3 is particularly suitable to be used as calibrator for the above mentioned DPP3-specific assays that were so far calibrated with the use of BCL dilutions and recombinant his-DPP3 [18]. The use of purified human DPP3 as calibrator allows precise batch-to-batch purity and activity assessment and reduces biological material risk management. The recalibration using purified material additionally offers the possibility of extending the assay detection range in lower range, increasing its sensibility. Although the normal distribution of DPP3 levels in the plasma in healthy individuals is approximately 58.6 U/L [18], other body fluids may have a naturally reduced DPP3 content, which can be better assessed with increased assay sensibility.

## Supporting information

S1 Fig Mass spectrometric molecular weight determination of DPP3 via HPLC-ESI-MS. (A) Summed ESI-MS Spectra of hDPP3. (B) Charge-state deconvoluted spectrum of hDPP3. (C) Zoom view into charge-state deconvoluted spectrum of hDPP3 (B).
(TIF)

## Abbreviations

BCL: blood cell lysate
BSA: bovine serum albumin
DPP: dipeptidyl peptidase
DSF: differential scanning fluorimetry
ECA: enzyme capture assay
IAP: immune-affinity purification
IEX: ion exchange
LIA: luminescent immuno assay
PBS: phosphate buffer saline
SAA: soluble activity assay
SDS-PAGE: sodium dodecyl sulphate polyacrylamide gel electrophoresis
SEC: size exclusion chromatography
