# Supplementary figures and images for: A novel and highly efficient purification procedure for native human dipeptidyl peptidase 3 from human blood cell lysate
Source: PLoS One. 2019 Aug 7;14(8):e0220866. doi: 10.1371/journal.pone.0220866 (PMC6685676; doi:10.1371/journal.pone.0220866)

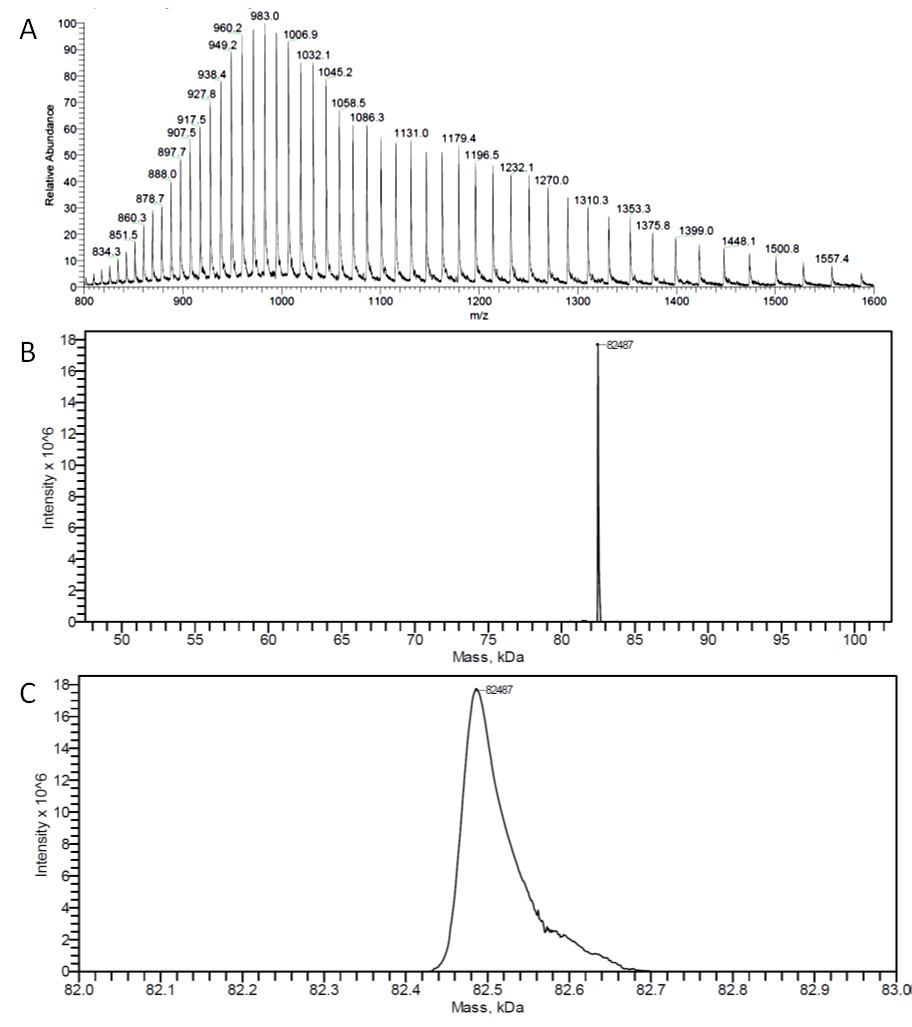

Supplement: S1 Fig — (A) Summed ESI-MS Spectra of hDPP3. (B) Charge-state deconvoluted spectrum of hDPP3. (C) Zoom view into charge-state deconvoluted spectrum of hDPP3 (B). (TIF) [file pone.0220866.s001.tif]
